# Knowledge, perception, and clinical experiences on molar incisor hypomineralization amongst Portuguese dentists

**DOI:** 10.1186/s12903-022-02284-1

**Published:** 2022-06-22

**Authors:** Rita Maria Delgado, João Botelho, Vanessa Machado, José João Mendes, Luísa Bandeira Lopes

**Affiliations:** 1 Clinical Research Unit, Centro de Investigação Interdisciplinar Egas Moniz, Egas Moniz – Cooperativa de Ensino Superior, CRL, 2829-511 Almada, Portugal; 2 Evidence-Based Hub, Centro de Investigação Interdisciplinar Egas Moniz, Egas Moniz – Cooperativa de Ensino Superior, CRL, 2829-511 Almada, Portugal

**Keywords:** Molar incisor hypomineralization, Perception, Clinical experiences, Developmental defects, Dentists, Oral health

## Abstract

**Aim:**

Molar incisor hypomineralization (MIH) is a prevalent oral health condition whose knowledge by dentists is key to the best clinical outcome. This study aimed to evaluate the knowledge, perceptions and clinical experiences of MIH among Portuguese dentists.

**Methods:**

A cross-sectional structured questionnaire was distributed nationally through a web-based survey platform. Data concerning demographic variables, years of experience, dental specialty, MIH prevalence, diagnosis, severity, training demands and clinical management of MIH were collected. We calculated a knowledge score (KS), and compared data between Pediatric Dentists (PDs), General Dental Practitioners (GDPs) and other dental specialties (ODS).

**Results:**

Overall, 2.2% of Portuguese dentists (n = 257) answered the questionnaire. Most participants reported having identified MIH in their practice (82.5%), with PD reporting the prevalence appeared to have increased, and practically all (91.7%) considered it a public health problem. Resin composite was often the used material to restore MIH teeth (56.0%), however PDs indicated glass ionomer cements as the preferred and preformed crowns a better option. The average KS on MIH was 41.3 (± 5.7), with GDPs having a similar score than PDs. Most respondents (94.9%) reported a lack of information about MIH and were willing to receive appropriate clinical training.

**Conclusions:**

The average knowledge on MIH was considered low among Portuguese dentists. Respondents perceived an increased incidence of MIH, despite the lack of prevalence data in Portugal. The material of choice was Glass Ionomer and performed crowns, by PDs, while GDPs and ODS reported poor confidence to manage MIH. These results may serve future programs to increase knowledge, perceptions and clinical experiences towards MIH.

**Supplementary Information:**

The online version contains supplementary material available at 10.1186/s12903-022-02284-1.

## Introduction

The report of opacities in the first permanent molars, since the late 1970s, has been observed and analyzed, yet only in 2001, the European Academy of Paediatric Dentistry (EAPD) firstly proposed the term “molar incisor hypomineralization” (MIH) [1, 2]. Thenceforward, several lines of evidence focused on MIH diagnosis, prevention and clinical management [3].

Presently, MIH is an increasingly common dental defect in clinical practice. Globally, its estimated worldwide prevalence is 13.5%, with affected incisors in 36.6% of the cases [4], and about 27.4% of cases require therapeutic interventions [5]. Clinically, MIH is characterized by the translucency of enamel with a white, yellow to brownish coloration depending on severity [3, 6–9]. As a consequence, hypomineralized enamel leads to post-eruptive breakdown and hypersensitivity, and it is prone to the development of carious lesions and pain [3, 6, 8–10].

As a condition defined in the beginning of the century, the perception and education of clinicians regarding MIH has been a research topic of interest [11–18]. Several studies assessed the knowledge, perceptions, and clinical experiences towards multiple countries [11–18]. In the main, these reports frequently show a need for continuing education and dissemination of the latest best evidence on MIH. In Portugal, such research has never been conducted, and ascertaining such indicators becomes relevant in what public health and education concern. Herein, we conducted a questionnaire-based survey to assess and compare the knowledge, perception, and clinical experience towards MIH between the pediatric dentists (PDs), general dental practitioners (GDPs) and other dental specialties (ODSs) in Portugal.

## Material and methods

### Setting and participants

The study population was dentists registered in the Portuguese Dental Association (PDA). A structured questionnaire was distributed nationally via Google Forms, a web-based survey platform, between May and June 2021. The questionnaire was also made available in social networks (such as Facebook and Instagram) to increase the number of participants. The following inclusion criteria were set in this study: (1) registered in the Portuguese Dental Association, (2) practicing dental medicine, (3) willing to participate and complete the survey. Participants were asked to complete the online questionnaire on their own time.

### Questionnaire

The questionnaire from Gambetta-Tessini et al. [12] modified by Gamboa et al. [18] was used to assess the knowledge, perceptions and clinical experiences towards MIH among dental practitioners in Portugal. The questionnaire consisted of three main sections according with Gamboa et al. [18].

In the first section, the participants reported sociodemographic information including sex, age, years of dental practice (< 5; 5 to 10, 11 to 20 or more than 21 years of dental practice) and qualifications (Doctor of Dental Surgery [DDS], Master of Science [MSc], Doctor of Philosophy [PhD], Post-graduate degree, Specialty). Specific modifications to the Portugal panorama were included for additional qualifications and training (Table 1). Within qualifications, participants were classified as GDPs, PDs or other dental specialties (ODS).

**Table 1:** Demographic characteristics of the study participants

Characteristics	Total N = 257 n (%)	GDPs N = 130 n (%)	PD N = 24 n (%)	ODS N = 103 n (%)	*p*-value
*Sex*					
Female	174 (67.7)	98 (75.4)	24 (100.0)	52 (50.5)	< 0.001
Male	83 (32.3)	32 (24.6)	0 (0.0)	51 (49.5)	
*Age group*					
≤ 30	101 (39.3)	71 (54.6)	6 (25.0)	24 (23.3)	< 0.001
31–40	69 (26.8)	19 (14.6)	10 (41.7)	40 (38.8)	
41–50	75 (29.2)	36 (27.7)	6 (25.0)	33 (32.0)	
≥ 51	12 (4.7)	4 (3.1)	2 (8.3)	6 (5.8)	
*Years of practice*					
< 5	70 (27.2)	56 (43.1)	2 (8.3)	12 (11.7)	< 0.001
5–10	70 (27.2)	30 (23.1)	8 (33.3)	32 (31.1)	
11–20	90 (35.0)	35 (26.9)	10 (41.7)	45 (43.7)	
> 21	27 (10.5)	9 (6.9)	4 (16.7)	14 (13.6)	
*Degree level*					
DDS/MSc/PHD	112 (43.6)	64 (24.9)	6 (2.3)	42 (16.3)	< 0.001
Post-graduation + MSc/PhD	122 (47.5)	63 (24.5)	8 (3.1)	51 (19.8)	
Specialty + MSc/PhD	32 (12.5)	3 (1.2)	14 (5.4)	15 (5.8)	

*DDS* Doctor of Dental Surgery, *GDP* General Dental Practitioner, *MSc* Master of Science, *PD* Pediatric Dentist, *PhD* Doctor of Philosophy, *ODS* Other Dental Specialties

Statistically significant (*p* < 0.05), Pearson’s chi-square (χ2) test

In the second section, participants were asked about their knowledge of possible aetiological factors, period of occurrence and the caries pattern seen in MIH. The knowledge was assessed through the KS proposed by Gambetta-Tessini et al. [12], that is fully described in the Statistics section.

Lastly, the third section accounted for participants’ perceptions, clinical experience and practitioners’ confidence in diagnosing and treating MIH, along with preferences in regards to continuing education and views on the necessity for clinical training regarding MIH. The treatment options were: microabrasion; infiltrating resin; glass ionomer; composite; amalgam; preformed crowns; and, extraction. As there is no MIH prevalence reference score for Portugal, we used a reference value from Schwendicke et al. 2018 [5]. The response distribution among the different answers provided information to assess the participants’ perception and clinical experience components.

The questionnaire was pilot-tested among 15 volunteer dentists at a 1:1:1 ratio for PD:GDP:ODSs (five each), 9 women and 6 men (27–49 years old), with a diversity of background and with < 5 years up to 20 years of experience, to ensure its applicability. We then made some revisions to the original questionnaire according to the instructions and opinions provided by the pilot tested volunteers (minor Portuguese language changes), and the final translated Portuguese version was applied via Google Forms, as previously reported.

### Statistics

Data was automatically entered into an Excel spreadsheet by Google Forms. Data was analysed using R software. Descriptive statistics (frequencies, percentage, mean) were determined. After confirming the non-existence of data normality and homoscedasticity through the Shapiro–Wilk test, three groups (GDPs, PDs and ODSs) were compared based on their socio-demographic backgrounds, specialties, and practice profiles. Continuous variables were compared using Kruskal–Wallis test, while for categorical variables we employed chi-square tests.

A KS was computed based on the answers in the knowledge section of the questionnaire. The scoring method was adapted from the original questionnaire [12] (detailed in Additional file 1: Annex S1). Following the proposed classification scheme, all ten-answer scores resulted in a single continuous variable assigned as the KS for the participant (ranging from a minimum score of 20 to a maximum score of 60). The level of significance was set at 5% for all inferential analyses.

## Results

### Characteristics of the participants

Among 11,640 dentists registered in the PDA, 257 answered the questionnaire (2.21%). Of the total number of participants, 50.5% were GDPs, 9.3% were PDs and 40.0% belonged to ODSs (Table 1). About 39.3% were 30 years or lower, and 35.0% (n = 90) had practiced for 11 to 20 years (Table 1).

### Knowledge of MIH

Concerning the prevalence of MIH in Portugal perceived by the Portuguese dentists, 32,7% of these consider that the prevalence is between 5 and 10%.

The total KS was 41.2 (± 5.7), without significant differences between PDs (41.5 ± 5.7), GDPs (41.9 ± 6.5) or ODSs (40.5 ± 3.6) (*p* = 0.214). These results confirmed that PDs did not present superior knowledge regarding MIH, as it should be expected (Table 2).

**Table 2:** Percentage distribution of knowledge scores of GDPs, pediatric dentists and other dental specialists for each question regarding MIH knowledge

Questions	Total N = 257 N (%)	GDPs N = 130 N (%)	PDs N = 24 N (%)	ODSs N = 103 N (%)	*p*-value
*It is known that MIH is a developmental defect that differs from amelogenesis and hypoplasia?*					
Yes	234 (91.1)	119 (91.5)	24 (100.0)	91 (88.3)	0.190*
No	23 (8.9)	11 (8.5)	0 (0.0)	12 (11.7)	
*What is the prevalence of MIH in Portugal?*					
< 5%	38 (14.8)	18 (13.8)	1 (4.2)	19 (18.4)	0.163*
5–10%	84 (32.7)	38 (29.2)	6 (25.0)	40 (38.8)	
10–20%	44 (17.1)	25 (19.2)	6 (25.0)	13 (12.6)	
> 20%	38 (14.8)	18 (13.8)	1 (4.2)	19 (18.4)	
Not sure	61 (23.7)	31 (23.8)	6 (25.0)	24 (23.3)	
*Which of the following might be the etiology of MIH:*					
Genetic factors	143 (55.6)	81 (62.3)	7 (29.2)	55 (53.4)	0.1875*
Environmental factors	89 (34.6)	44 (33.8)	8 (33.3)	37 (35.9)	
Acute medical conditions affecting the hand or child	93 (36.2)	55 (42.3)	7 (29.2)	31 (30.1)	
Chronic medical condition affecting mother and child	79 (30.7)	39 (30.0)	6 (25.0)	34 (33.0)	
Antibiotics or medications	94 (36.6)	50 (38.5)	5 (20.8)	39 (37.9)	
Exposure to fluoride	29 (11.3)	18 (13.8)	0 (0.0)	11 (10.7)	
Unknown etiology	133 (51.8)	72 (55.4)	18 (75.0)	43 (41.7)	
*What is the period/duration that this occurrence can happen?*					
During the pregnancy	46 (17.9)	25 (19.2)	3 (12.5)	18 (17.5)	0.476*
First year of life	26 (10.1)	16 (12.3)	0 (0.0)	10 (9.7)	
Third year of life	12 (4.7)	8 (6.2)	0 (0.0)	4 (3.9)	
Pregnancy up to the first year of life	71 (27.6)	34 (26.2)	9 (37.5)	28 (27.2)	
Pregnancy up to the third year of life	102 (39.7)	47 (36.2)	12 (50.0)	43 (41.7)	
*In your opinion, do you think the pattern of caries related to MIH is different from the classic caries pattern?*					
Yes	228 (88.7)	117 (90.0)	23 (95.8)	88 (85.4)	0.385*
No	10 (3.9)	3 (2.3)	1 (4.2)	6 (5.8)	
Not sure	19 (7.4)	10 (7.7)	0 (0.0)	9 (8.7)	
Knowledge score, mean (SD) [min–max]	41.3 (5.7) [21–60]	41.9 (6.0) [21–60]	41.5 (5.7) [35–46]	40.5 (3.6) [25–52]	0.214**

*GDPs* General Dental Practitioners, *PDs* Pediatric Dentists, *ODSs* Other Dental Specialties

*Statistically significant (*p* < 0.05), Pearson’s chi-square (χ2) test

**Statistically significant (*p* < 0.05), Kruskal–Wallis test

### Perceptions, clinical experience, and continuing education

Regarding the cases found in clinical practice, all PDs reported to have cases of MIH. Concerning which type of dental development defect they discovered most frequently in their clinical practice, the GDPs were faced with Yellow/brown opacities (34.6%) and, in turn PDs with Yellow/brown opacities (20.8%), post-eruptive fractures and Yellow/brown opacities (20.8%) and White opacities and Yellow/brown opacities (29.2%) (*p* < 0.001). Towards other permanent teeth with MIH, 72% of all participants state that they did not find these defects in other permanent teeth, being 87,5% PDs (*p* = 0438). Yet, 83,3% of PDs diagnosed these defects in primary second molars (*p* < 0.001).

Most PDs (95.8%) perceived that the incidence of MIH has been increasing in Portugal, although 49% of all participants do not agree with this idea. Concerning the type of defect, and excluding PDs, 82.9% of participants considered referring to a PD for a differentiated treatment option. Towards preventive treatment, 62,3% of all respondents considered Fluor varnish, being that 66,7% PDs (*p* < 0.001). Regarding the MIH treatment, composite restoration is the most used option by 56% of all the participants. In turn, PDs preferred Glass Ionomer as well as preformed crowns (75%, 70,8% respectively), and note that none of all respondents have chosen the use of amalgam (*p* < 0.001). In the treatment of MIH, 52.5% of all participants feel little confidence, being 53.1% GDPs and 61.2% ODSs. Nevertheless, 58.3% of PDs feel trusting. Most dentists questioned (94.9%), reported that they do not receive information about MIH, with 70.8% being PDs (*p* < 0.001). Nevertheless, GDPs were willing to receive more information regarding MIH diagnosis, etiology, and treatment, while only 6.2% of the questioned participants, and 8.3% being PDs, reported that receiving such information was not relevant (*p* < 0.048) (Table 3).

**Table 3:** Perceptions, clinical experience, and continuing education aspects of GDPs, Pediatric Dentists and Other Dental Specialists regarding MIH

Questions	Total N = 257 N (%)	GDPs N = 130 N (%)	PDs N = 24 N (%)	ODSs N = 103 N (%)		*p*-value
*In your clinical practice, do you find cases of MIH?*						
Yes	212 (82.5)	104 (80.0)	24 (100)	84 (81.6)		0.0574
No	45 (17.5)	26 (20.0)	0 (0.0)	19 (18.4)		
*What is the most common type of dental development defect that you find in your clinical practice?*						
White opacities	50 (19.5)	30 (23.1)	2 (8.3)	18 (17.5)		< 0.001
Yellow/brown opacities	82 (31.9)	45 (34.6)	5 (20.8)	32 (31.1)		
Post-eruptive fractures	4 (1.6)	1 (0.8)	2 (8.3)	1 (1.0)		
None	18 (7.0)	9 (6.9)	0 (0.0)	9 (8.7)		
Post-eruptive fractures and Yellow/brown opacities	13 (5.1)	1 (0.8)	5 (20.8)	7 (6.8)		
White opacities and Yellow/brown opacities	78 (30.4)	40 (30.8)	7 (29.2)	31 (30.1)		
White opacities. Yellow/brown opacities and Post-eruptive fractures	12 (4.7)	4 (3.1)	3 (12.5)	5 (4.9)		
*What other permanent teeth have you found MIH defects in?*						
Premolars	7 (2.7)	3 (2.3)	1 (4.2)	3 (2.9)		0.438
Second permanent molars	45 (17.5)	31 (23.8)	2 (8.3)	12 (11.7)		
Canines	5 (1.9)	2 (1.5)	0 (0.0)	3 (2.9)		
Canines and Premolars	1 (0.4)	0 (0.0)	0 (0.0)	1 (1.0)		
Premolars and Second permanent molars	9 (3.5)	5 (3.8)	0 (0.0)	4 (3.9)		
Canines and Second permanent molars	5 (1.9)	3 (2.3)	0 (0.0)	2 (1.9)		
None	185 (72.0)	86 (66.2)	21 (87.5)	78 (75.7)		
*Do you notice these defects in deciduous second molars?*						
Yes	95 (37.0)	42 (32.3)	20 (83.3)	33 (32.0)		< 0.001
No	162 (63.0)	88 (67.7)	4 (16.7)	70 (68.0)		
*Do you have any idea if the incidence of MIH has been increasing?*						
Yes	131 (51.0)	63 (48.5)	23 (95.8)	45 (43.7)		< 0.001
No	126 (49.0)	67 (51.5)	1 (4.2)	58 (56.3)		
*In case you identify a defect of this type (MIH), would you refer it to a pediatric dentist for differentiated treatment?*						
Yes. or when possible	213 (82.9)	98 (75.4)	–	94 (91.3)		< 0.001
No	44 (17.1)	32 (24.6)	–	9 (8.7)		
*In your opinion, does MIH represent a public health problem similar to tooth decay?*						
Yes	195 (75.9)	95 (73.1)	22 (91.7)	78 (75.7)		0.148
No	62 (24.1)	35 (26.9)	2 (8.3)	25 (24.3)		
*What type of preventive treatment do you usually apply for these teeth?*						
Fluor varnish	160 (62.3)	93 (71.5)	16 (66.7)	51 (49.5)		< 0.001
Diamino-fluoride of silver	13 (5.1)	4 (3.1)	1 (4.2)	8 (7.8)		
CCP-ACP or cracked amorphous casein phosphate	44 (17.1)	10 (7.7)	15 (62.5)	19 (18.4)		
Crack Sealant	100 (38.9)	58 (44.6)	3 (12.5)	39 (37.9)		
Others	66 (25.7)	31 (23.8)	5 (20.8)	30 (29.1)		
*What type of treatment do you usually apply for MIH?*						
Microabrasion	47 (18.3)	24 (18.5)	6 (25.0)	17 (16.5)		< 0.001
Infiltrating resin	86 (33.5)	51 (39.2)	5 (20.8)	30 (29.1)		
Glass ionomer	97 (37.7)	48 (36.9)	18 (75.0)	31 (30.1)		
Composite	144 (56.0)	71 (54.6)	14 (58.3)	59 (57.3)		
Amalgam	3 (1.2)	0 (0.0)	0 (0.0)	3 (2.9)		
Preformed crowns	73 (28.4)	27 (20.8)	17 (70.8)	29 (28.2)		
Extraction	6 (2.3)	1 (0.8)	3 (12.5)	2 (1.9)		
*In your opinion, do you feel confident in diagnosing MIH?*						
Very confident	20 (7.8)	5 (3.8)	10 (41.7)	5 (4.9)		< 0.001
Trusting	129 (50.2)	66 (50.8)	13 (54.2)	50 (48.5)		
Little confident	90 (35.0)	47 (36.2)	1 (4.2)	42 (40.8)		
Not at all confident	18 (7.0)	12 (9.2)	0 (0.0)	6 (5.8)		
*In your opinion, do you feel confident in managing your MIH treatment?*						
Very confident	14 (5.4)	2 (1.5)	7 (29.2)	5 (4.9)		< 0.001
Trusting	76 (29.6)	39 (30.0)	14 (58.3)	23 (22.3)		
Little confident	135 (52.5)	69 (53.1)	3 (12.5)	63 (61.2)		
Not at all confident	32 (12.5)	20 (15.4)	0 (0.0)	12 (11.7)		
*Do you receive any information about MIH?*						
Yes	13 (5.1)	3 (2.3)	7 (29.2)	3 (2.9)		< 0.001
No	244 (94.9)	127 (97.7)	17 (70.8)	100 (97.1)		
*Would you like to know more about Hypomineralization?*						
Diagnosis	178 (69.3)	95 (73.1)	15 (62.5)	68 (66.0)		0.048
Etiology	171 (66.5)	84 (64.6)	16 (66.7)	71 (68.9)		
Treatment	228 (88.7)	123 (94.6)	21 (87.5)	84 (81.6)		
No	16 (6.2)	2 (1.5)	2 (8.3)	12 (11.7)		

*GDPs* General Dental Practitioners, *MIH* molar incisor hypomineralization, *PDs* Pediatric Dentists, *ODSs* Other Dental Specialties

Statistically significant (*p* < 0.05), Pearson’s chi-square (χ2) test

## Discussion

This is the first survey exploring the knowledge, perception and clinical experience on MIH among Portuguese dentists. Overall, the knowledge on MIH was considered low (average of 41.3 on a scale of 20 to 60), while perception and clinical experience were different between PDs and non-PDs practitioners (GDPs and ODSs). In general, around 75% of the respondents view MIH as a public health problem, only PDs reported to be confident diagnosing and managing MIH cases. In addition, most respondents (overall 94.9%, PDs 70.8%) did not received information about MIH and the majority would be interested in receiving more information on diagnosis, etiology and treatment, showing the need to implement continuing education in the diagnosis and treatment of MIH.

Overall, the average KS from the present study was lower than other counterparts from Australia [12], Chile [12] and Hong Kong [18]. This score proposed by Gambetta-Tessini [12] allows the comparison of knowledge among different groups (either geographic location or dental specialties) as well monitoring its evolution along with education measures (such as, continuing education programs or improvements to curricular contents of Pediatric Dentistry regarding MIH). These results may pave the way for the need to critically appraise both the curricular content among Portuguese dental schools (in undergraduate and specialty graduate programs) and cutting-edge unique courses to meet needs throughout the career.

Similarly to the reports from Australia, Chile and Hong Kong [12, 18], all PDs reported to have found MIH cases in clinical practice. Furthermore, although PDs did report being aware of all phenotypes of MIH (in molars, incisors or premolars), they were not aware of canine-affected MIH cases [19]. Likewise, when asked if MIH was found in primary second molars, highly reported in literature [9, 20], the majority of GDPs and ODSs reported to have not observed such cases, as well as a remarkable 16.7% of PDs.

Considering the latest estimated MIH global prevalence of 13.5% [4], the cross-country variety may range from 2.8 to 40.2% [21]. The prevalence of MIH in Portugal has never been estimated, therefore we used the worldwide estimated result of 13.5%, yet 32.7% of the participants believe that this may range between 5 and 10%. From the PDs view, the prevalence of MIH has been increasing, in line with the views from Spanish counterparts [11].

In what the etiology of MIH concerns, the majority of PDs (75%) report to be unknown, contrasting, for example, with New Zealand counterparts that attribute the etiology to medical conditions because of their great experience on MIH cases in populations with compromised health [13]. In addition, surveys from Hong Kong, Iraq, Australia and Chile report that chronic and acute conditions that affect the mother and child are the most relevant etiological factors of MIH [12, 16, 18]. Considering that the etiology of MIH is still unknown, possibly multifactorial, and far from fully understood, these variations are understandable and warrant a particular focus on future research.

Most GDPs reported yellow or brown opacities as the major manifestations of MIH defects mostly found in their clinical practice, similarly to reports from Australia, Spain and Hong Kong [11, 13, 18]. PDs simultaneously reported white, yellow and brown opacities and post-eruptive fractures as the most common findings, showing a relatively higher awareness of the diversity of MIH phenotypes [11, 18]. GDPs showed a lack of recognition ability towards MIH diagnosis, particularly regarding opacities or post-eruptive fractures. Despite this, both PDs and GDPs attested knowing that MIH and other dental developmental defects differ, namely amelogenesis and hypoplasia. Surveys carried out in the United Kingdom, Spain and Malaysia, showed difficulties from dentists in distinguishing MIH from other enamel defects [11, 15, 17].

In what treatment of MIH concerns, PDs reported glass ionomer (75.0%), preformed crown (70.8%) and composite resins (58.3%) as the preferred treatment options. These results contrasted with the reports from GDPs and ODSs, that reported to prefer composite resins (54.6% for GDPS and 57.3% for ODSs), infiltrating resins (39.2% for GDPs and 29.1% for ODSs), and glass ionomer (36.9% for GDPS and 30.1% for ODSs). The variety of materials used in the treatment of teeth affected by MIH possibly denotes the lack of evidence-based guidelines, excluding composite resins that are recommended in moderate MIH lesions [11, 19]. Therefore, further research on the physical properties and clinical performance of restorative materials is recommended to fill this gap of knowledge [13]. The persistence of absent clinical decision-trees and best evidence consensus regarding restorative materials will contribute to enduring difficulties in the clinical management of MIH teeth. Although there are general indications on which professionals can rely, perhaps the lack of dissemination and information is reflected in the use of different materials for the treatment of teeth with MIH, which was observed in this study. Comparing these results with other countries, Australian and Chilean dentists reported glass ionomer as the treatment of choice [12, 19], in Spain the resin-modified glass ionomer cement and composite resins were preferred [11]. Particularly in post-eruptive fractures, surveys from Hong Kong and New Zealand reported preformed crowns as the treatment of choice [13, 18]. According to Elhennawy and Schwendicke, glass ionomer and amalgam restorations have a higher failure rate, opposite to the higher successful rates of composite restorations and preformed crowns [22].

About the received information on MIH, the results point to a gap in the training of medical dentists. Similarly to Portugal, dentists from Malaysia and Hong Kong also reported that they did not receive information about MIH [17, 18]. Spanish and Hong Kong GDPs received less training than PDs. However, Australian and Chilean reported to have received more information about MIH, which was conveyed to have increased their awareness and knowledge [11–13, 18]. Access to information is essential to enhance an early diagnosis of MIH and adequate patient monitoring. This not only allows for the application of preventive measures to minimize post-eruptive sensitivity and fractures, but also allows a strict control of the affected teeth [18]. Therefore, the availability of information on MIH is key to a future coordinated public health response with a multidisciplinary engagement from all specialties in Dentistry.

As such, these results may be the first line of evidence on the level of PDs, GDPs and other specialists regarding this condition, and what gaps of knowledge and opportunities are needed to be filled in the upcoming years in Portugal. Nevertheless, this study presented a low response rate, which may limit the generalizability to the whole country.

## Conclusions

The knowledge on MIH was overall low and PDs reported higher self-confidence on its clinical management. The perception and clinical experience towards MIH seems adequate, however participants self-reported the need for more professional continuing education.

## Supplementary Information

**Additional file 1**: MIH knowledge scoring according to Gambetta-Tessini et al. (2016).

## Data Availability

The data are placed on the Zenodo.org platform with https://doi.org/10.5281/Zenodo.5815404, with open access.
