# Supplementary material for: Knowledge, perception, and clinical experiences on molar incisor hypomineralization amongst Portuguese dentists
Source: BMC Oral Health. 2022 Jun 22;22:250. doi: 10.1186/s12903-022-02284-1 (PMC9219218; doi:10.1186/s12903-022-02284-1)
Supplement: Supplementary file 1 — Additional file 1: MIH knowledge scoring according to Gambetta-Tessini et al. (2016). [file 12903_2022_2284_MOESM1_ESM.docx]

**Annex 1.** MIH knowledge scoring according to Gambetta-Tessini et al. (2016).

| Question | | If Answered **Yes** | If Answered **No** |
| --- | --- | --- | --- |
| 1) Have you been aware that MIH is a developmental defect that differs from fluorosis and hypoplasia? | | 9 | 0 |
| 2) How prevalent do you think MIH might be in your community? (One option chosen) | <5% | 1 | NA^#^ |
|  | 5-10% | 1 | NA^#^ |
|  | 10-20% | 6 | NA^#^ |
|  | >20% | 1 | NA^#^ |
|  | Not sure | 0 | NA^#^ |
| 3–8) Do you think they are involved in the aetiology of MIH? | 3) Genetic factors | 5 | 4 |
|  | 4) Environmental contaminants | 5 | 4 |
|  | 5) Chronic medical conditions affecting mother or child | 6 | 3 |
|  | 6) Acute medical conditions affecting mother or child | 6 | 3 |
|  | 7) Antibiotics or medications | 5 | 4 |
|  | 8) Fluoride exposure | 1 | 8 |
| 9) During what time/period do you think this insult occurs? (One option chosen) | During pregnancy | 1 | NA^#^ |
|  | 1^st^ year of life | 3 | NA^#^ |
|  | 3^rd^ year of life | 0 | NA^#^ |
|  | Pregnancy to 1^st^ year of life | 3 | NA^#^ |
|  | Pregnancy to 3^rd^ year of life | 2 | NA^#^ |
| 10) Do you think the pattern of caries related to MIH is different from the classical caries pattern? | | 7 | 1 (no) or 1 (not sure) |

^#^ Answer “No” does not apply because it is a single-choice question.
